# Are protected characteristics associated with mental health care inequalities in the adult UK general population? a cross-sectional study

**DOI:** 10.1371/journal.pone.0308279

**Published:** 2024-08-06

**Authors:** Claire Wicks, Cara Booker, Meena Kumari, Antonella Trotta, Susan McPherson

**Affiliations:** 1 School of Health and Social Care, University of Essex, Colchester, United Kingdom; 2 Institute for Social and Economic Research, University of Essex, Colchester, United Kingdom; University of Toronto, CANADA

## Abstract

This study investigates the association between protected characteristics and inequalities in mental health care in the UK. Multinomial regression was used to model the association between protected characteristics and self-reported distress. Data was extracted from waves 6–10 (2014–2019) of the UK Household Longitudinal Study. Two risk categories were constructed: “undiagnosed distress” referred to a General Health Questionnaire-12 (GHQ-12) score above “caseness” along with no history of mental health diagnosis; “diagnosis without self-report symptoms” referred to a GHQ-12 score consistently below “caseness” within the study time frame but having received a mental health diagnosis. Compared to people without a disability, people with a disability are at considerably greater risk of both undiagnosed distress (Relative risk ratios (RRR) 2.76; Confidence Interval (CI): 2.55, 2.99) and diagnosis without self-reported symptoms (RRR 3.61; CI: 2.80, 4.66). Likewise, women were more likely than men to report undiagnosed distress (RRR = 1.49; CI: 1.38,1.61) or a diagnosis without self-reported symptoms (RRR = 1.38; CI: 1.08, 1.76. Lesbian, gay, and bisexual people are at greater risk of undiagnosed distress compared with heterosexual people (RRR 1.42; CI: 1.19, 1.70). Adults aged 16–24 years were at greatest risk compared to all other age groups. People from a minority ethnic background had a reduced risk of diagnosis without self-report symptoms compared with people from a White ethnic background (RRR 0.34; CI: 0.20, 0.61). Education, employment and income variables moderated some of these associations. This is the first study to examine diagnosis without self-report symptoms alongside undiagnosed distress. Findings suggest that addressing inequality in mental health care requires increased understanding of the needs and strengths within different groups and to provide appropriate forms of social, medical or psychosocial intervention rather than a singular focus on increasing detection, diagnosis and treatment. People with a disability appear to be at greatest disadvantage, requiring greater attention in policy and practice.

## Introduction

According to the Adult Psychiatric Morbidity Survey (APMS), approximately one in six adults in England experience symptoms of a common mental disorder (CMD), such as depression or anxiety, at any one time. Less than half of the those who experience severe symptoms of CMDs (“requiring intervention”) report being in receipt of any treatment (medical or psychological) [1]. Moreover, only a third with CMDs warranting “primary care recognition” had ever received a diagnosis, suggesting that not all those in the community needing mental health support are being identified by mental health services and offered support. Analyses of APMS data also indicate inequalities in mental health with higher prevalence of CMDs among Black women, adults under the age of 60 who live alone, women living in large households, adults not in employment, those in receipt of benefits (in particular benefits associated with disability), and those who smoke cigarettes [1]. While these analyses provide evidence of a gap between experience of symptoms and accessing care, and evidence of inequalities in the occurrence of poor mental health, the APMS data do not provide detailed analysis of inequalities in access to mental health care across all protected characteristics. There have been intermittent commitments from the United Kingdom (UK) Government over several decades to increase funding for mental health services, most recently in 2019 [2] but there is no consistent indication of funding being targeted to address inequalities in access to care and this may in part be due to a lack of reliable analysis of what broad inequalities in access to mental health care are currently present in the UK.

Access refers to the “availability of services that are timely, appropriate, easy to get to and use, and sensitive to user choice and need” [3]. Inequalities in access to mental health care have been identified as impacting on various groups within the population and can often lead to exacerbation of difficulties, including ethnic minority groups, women, sexual minorities, people with disabilities, and older people. For example, a review of inequalities in healthcare by ethnic group commissioned by the National Health Service (NHS) Race and Health Observatory reports a series of barriers experienced by ethnic minority people when accessing mental health services [4]. Barriers included mental health professionals perceived as ‘patronising and judgemental’, failing to listen to concerns, and lack of understanding about racism or how it impacts on mental health. There is also evidence that compared with people from a White British background some ethnic minority groups are more likely to be detained under the Mental Health Act and that racism and discrimination play a role in this [5,6].

Similarly, evidence suggests that poor access to appropriate services can result in women being unjustly detained in secure settings and allocated to units outside of their home region [7]. Concerns about having a child removed or being seen as an unfit parent can prevent women from seeking support and experiences with mental health services can be disempowering or retraumatising for women who have previously experienced trauma or discrimination [8]. The same report also found women experience lack of voice and control over many aspects of their mental health treatment. Lesbian, gay and bisexual (LGB) groups often experience internalised stigma and may be reluctant to seek support as a result of a felt need to conceal their sexuality [9]. This is supported by findings from the UK’s National LGB survey which reported that 72% of respondents found it “had not been easy” to access mental health services [10]. Reasons included having to wait too long; anxiety or embarrassment about reporting symptoms; and because their General Practitioner (GP) was unsupportive. Twenty-two percent of respondents who accessed mental health services reported their experience as “mainly or completely negative” [10]. Furthermore, a survey of 5000 LGB and trans people in the UK revealed experiences of discrimination, lack of understanding of their specific health needs, and unequal treatment [11].

Analysis of data from the European Health Interview Survey found that compared to people without a disability, people with severe disability were 7.24 times more likely to have an unmet mental health need due to cost and 4.32 times more likely due to transportation issues [12]. Research conducted in the UK [13] found people with disabilities face barriers to accessing a GP, including surgeries enforcing on-the-day only appointments, meaning people with disabilities cannot always find a suitable carer to take them at short notice. The research also identified accessibility issues at surgeries and communication problems, such as a shortage of staff able to communicate using sign language [13]. Given GPs are normally gatekeepers for referrals to mental health services this is likely to have a subsequent impact on access to mental health care for disabled people.

Mental health care needs of individuals also change across the lifespan. Mental health needs of older adults differ from younger adults with “different mental disorders, different presentations, multiple co-occurring medical or physical health conditions, different care plan needs and different contexts” [14]. For example, older people with depression will often experience difficulties in concentration and memory, making a diagnosis of dementia more likely. Evidence suggests current mental health care services in the UK are not meeting the needs of older people, with figures indicating 85% of older people with depression receive no help from the NHS, and that older people are one fifth as likely as younger age groups to have access to talking therapies but six times as likely to be on medication [15].

There are also a range of wider determinants which can impact on access to mental health care, specifically the social, economic, and environmental conditions in which people live and these can intersect with personal characteristics, such as age and ethnicity. Wider determinants often occur simultaneously and can have a cumulative effect on mental health outcomes. For example, level of educational attainment is positively correlated with income, which may determine quality of housing, the neighbourhood in which one can afford to live, which in turn has an impact on health and mental health through access to health services, schools and other health related environmental factors, such as amount of pollution and green space [16–18]. Certain groups of people may face greater disadvantage, for example, people living with a disability experience barriers that prevent equivalent educational attainment compared to people without a disability, and are more likely to be unemployed or economically inactive [19]. As such, when exploring mental health care inequalities, considering the impact of wider determinants may help to uncover the mechanisms driving inequalities.

The UK government sets out four domains of health inequalities identifying the broad range of individual characteristics and societal factors that contribute to health inequalities; one of these domains is protected characteristics as defined by the Equality Act 2010 [20]. Protected characteristics include, sex (male or female), age, being married or in a civil partnership, ethnic group, religion or belief, disability, being pregnant or on maternity leave, and gender reassignment. Some county level governments (“councils”) in the UK are grouping residents by protected characteristics to assess health and wellbeing and for policy and agenda setting. The Equality Act 2010 is a UK law designed to prevent discrimination in contexts such as job recruitment, employment rights, access to education, health and other services.

The Equality Act does not clearly define which manifestation of each characteristic is “protected”, only that discrimination should not occur on the basis of the characteristic. In different settings or contexts, it is possible that different groups within a protected characteristic may be more routinely disadvantaged. In order to apply the Equality Act to access to mental health support it is necessary to distinguish between the characteristics (e.g. sex, ethnicity) and the specific group or people within that characteristic that may need most protection, or rather is currently most disadvantaged. Therefore, analysis of inequalities in access to mental healthcare according to protected characteristics could reveal which individuals are most disadvantaged due to their characteristics, and therefore may need more focus including increased funding to redress existing inequalities.

The aim of the current study is therefore to identify inequalities in mental health care in the UK according to protected characteristics. Our approach is to look at the issue from a population health perspective rather than a clinical context, given that (as noted earlier), UK community data indicates significant levels of diagnosable mental health conditions which have not been diagnosed in a clinical setting. Our study also considers agreement between self-ratings and diagnostic status in two ways. First, we consider the standard APMS approach to identifying people who report mental health symptoms but who have not been diagnosed with a mental health condition, which we refer to as undiagnosed distress (UD). Unlike previous studies that compare mental health needs across populations, our study advances this literature by examining the risk of self-reported distress without a diagnosis, which might suggest inadequate healthcare access (i.e., under-treatment). Second, we consider people with mental health diagnoses but without self-reported mental health symptoms in the previous few years, which we refer to as diagnosis without self-reported symptoms (DWS). To our knowledge, this category has not been examined before, but disagreement on this dimension is of interest because it might suggest over-medicalisation (i.e., over-treatment) of social conditions, like loneliness. Meaningful differences across populations in these statuses could provide insight into healthcare inequalities, which manifests as both under- and over-treatment. Given concerns about over-use of psychiatric medication [21], the potential over-medicalisation of some groups is another important aspect of inequality to map in the UK. The current study also aims to examine whether social and economic circumstances influence potential relationships between protected characteristics and risk of unmet need or diagnosis without symptoms. Using *Understanding Society*; the UK Household Longitudinal Survey (UKHLS), this study therefore examines first the relative risk of individuals having UD or DWS according to protected characteristic; and second the influence of social and economic variables in the association between protected characteristics and mental health care inequalities.

## Methodology

### Study design and participants

UKHLS [22] is a household panel study that includes more than 25,000 UK households. Original sample members were from the British Household Panel survey which was a randomly sampled UK population sample initially sampled in 1991 with sample boosts in 1999, 2001 and 2008. These sample members were transferred into the UKHLS study in 2009. New members were recruited to boost the sample in 2009, including an Ethnic Minority Boost sample and the addition of a range of other minority groups identified as being under-represented in the original sample. In 2015 a further sample boost took place focusing on increasing representation of immigrants and ethnic minorities. The Quality Profile of UKHLS (see www.understandingsociety.ac.uk/documentation/mainstage/quality-profile) provides full documentation on principles and procedures underlying study design; response rates for each wave; procedures for data processing, weighting and imputation; and profiles of response rates. Since 2009, each household member has been interviewed on an annual basis about a range of topics including health and wellbeing, employment, and finances. Interviews are carried out by trained interviewers or by self-completion online. Technical reports for each wave provide highly detailed information on recruitment and data collection methods (see www.understandingsociety.ac.uk/documentation/mainstage/technical-reports). These include details of the bespoke technical programming and software designed by Kantar, a world-leading provider of survey tools, marketing and data analytics. Details are provided of adaptations made for different participants including translations of all survey materials available for 9 of the most common languages used in the UK; a protocol for using agency translators or household members as translators for other languages. All interviewers working with translated versions of the survey pass an accreditation process detailed in the technical report. Interviewers receive extensive bespoke training for UKHLS including special procedures for working with translators; interviewing people in institutions; interviewing people with disabilities; and supporting participants to understand any difficult terms. There are further detailed protocols for proxy interviews where another adult in the household provides responses for participants who are unable to complete a full interview. The reports explain that where online surveys are incomplete, they may be completed in person and protocols are in place for reconciliation of data or duplication.

Data for this study was extracted from waves 6 to 10 (2014 to 2019) of the UKHLS main survey. Waves 11–13 (2020 to 2023) and wave 10 data collected after 2019 were excluded to ensure estimates were not skewed due to the impact of the Coronavirus pandemic on population level mental health [23]. During the pandemic, fluctuations in mental health were evident with worsening of disadvantage in some populations, including women and young adults [24]. Data for this study included the immigrant and ethnic minority boost sample added in wave 6. In addition, the study made use of a new question added in wave 10 that asked respondents whether they had ever been told by a doctor or health professional that they had an emotional, nervous, or psychiatric problem. In prior waves, respondents were only asked about history of clinical depression. Data from adult household members (aged 16+ years) who completed at least one wave of the UKHLS between 2005 and 2019 were included in the study.

### Measures

#### Demographic variables

Demographic variables that relate to protected characteristics as defined by the Equality Act 2010 were extracted from the UKHLS datasets for analysis. Full details of questionnaire modules, variables and response options are available at www.understandingsociety.ac.uk/documentation. For the purposes of the current analysis it was necessary to create dichotomous variables. Most questions in UKHLS have multiple response options. Most questions include the option “I don’t know” which was excluded. Most questions also include an “Other” response option which, where relevant, were incorporated as detailed below. The UK Equality Act includes sex as a protected characteristic which has categories male and female. This is mirrored in UKHLS which asks “Are you male or female?” with no alternative response options. This is because the question concerns sex and not gender, with sex (not gender) being a protected characteristic in the UK Equality Act. The analysis uses male as the reference group. Age was grouped into categories as per the UK Census’ recommended age categories, with 16–24 as the reference group and subsequent groups being 25–34, 35–44, 45–54, 55–64, 65–74, and 75+ [25]. This categorisation was chosen to ensure sufficient sample size of each age category for analysis and for identification of differences between life stages (e.g. young adulthood, middle age). Other protected characteristic variables were grouped into dominant and minority groups. Due to small sample sizes, minority groups could not be investigated independently. Marital status was categorised into either “married” or “in a civil partnership” (as the reference group) or not married or in a civil partnership (the latter including all other response options such as widowed, divorced, separated, never married, dissolved, or separated civil partnership). Disability was categorised into either no long-term health condition (the reference group) or having a long-term health condition. This was asked in the survey in a way that matched the UK Equality Act definition: “Do you have any long-standing physical or mental impairment, illness or disability” with response options being “Yes” or “No”. Long-standing is defined as something that has troubled the participant over a period of at least twelve months. Religion was categorised into no religious affiliation (the reference group), Christian (including Catholic), or a minority religion. Participants responding “Other” were grouped with minority religion. Ethnic group was either White British (the reference group) or ethnic minority (which included participants who selected “Any other ethnic group”). Sexual orientation was either heterosexual (the reference group) or LGB (people selecting Lesbian, Gay, Bisexual or Other were included in this category). Permission to access sexual orientation data was gained via a special licence application to the UK Data Service. Data about gender re-assignment was unavailable in the dataset. Pregnancy was not included due to the small number of respondents reporting a pregnancy in each wave.

#### Social and economic variables

Social and economic variables were included as covariates due to their relationship with mental health outcomes and inequalities. Variables included were individual net income, educational attainment (grouped as higher education as reference group, further education/professional education, high school or other); economic activity (grouped as employed as the reference group, unemployed, retired or other); and household size.

#### Measures of mental health

The 12-item General Health Questionnaire (GHQ-12) [26] is a self-report measure of non-specific mental distress during the past two weeks. The measure consists of 12 positively or negatively worded items (e.g., “Thinking of self as worthless”, “Able to concentrate”), with a 4-point Likert-type response scale (0 to 3). There are two ways of scoring the GHQ, Likert or caseness scoring. The Likert scale is a continuous measure of distress by summing the respondents scores on each item; whereas the caseness method uses a cut off score to determine whether individuals are cases or non-cases (i.e. likely to be experiencing psychological distress) [26]. The caseness method is widely used in population research, including for example, the Health Survey for England, and was chosen for this study since the design required participants to be divided into two categories based on their GHQ score. In the “caseness” scoring approach, response values are recoded as 0, 0, 1,1. The item scores are summed to give a scale running from 0 to 12. A score of four or above was used to indicate likelihood of a general psychiatric disorder [26]. The GHQ-12 has good sensitivity (83±4%) and specificity (76±3%) in general populations [27] and excellent reliability in the study sample (*α* = 0.91). Respondents’ GHQ-12 score reported at their most recent observation between waves 6 to 10 was used for analysis. Where GHQ-12 score was missing an average score was calculated.

#### History of mental health diagnosis

UKHLS asks respondents about history of diagnosed clinical depression in all waves and whether they have ever been diagnosed with an emotional, nervous or psychiatric problem or clinical depression in wave 10. The questions ask whether a doctor or other health professional has provided this diagnosis. The survey does not ask whether the diagnosis was from a public or private practitioner but the vast majority of people in the UK only have access to public-funded health services. Respondents who indicated history of a diagnosed mental health problem in either of these ways were treated as having a mental health diagnosis.

#### Mental health status

A *mental health status* variable was derived based on five possible combinations of GHQ-12 score and history of diagnosis. Three of the five combinations involved agreement between self-reported levels of distress (GHQ-12 caseness) and a history of professional diagnosis. The three forms of “Agreement” involved: 1. No evidence of psychological distress (GHQ-12 caseness score of between 0–3) and no history of mental health diagnosis; 2. Evidence of psychological distress (GHQ-12 caseness score of ≥ 4) and has a history of mental health diagnosis; 3. No current evidence of psychological distress (GHQ-12 caseness score of between 0–3) but has history of caseness and mental health diagnosis suggesting possible remission.

The research questions focus, however, on two statuses reflecting discrepancies between diagnosis and self-reported symptoms: 1. Evidence of psychological distress (GHQ-12 caseness score ≥ 4) but no history of mental health diagnosis (suggesting “Undiagnosed distress”, UD); 2. No evidence of psychological distress at any of the time points included (GHQ-12 caseness score of between 0–3 in all waves included) but has a history of mental health diagnosis (“Diagnosis without self-reported symptoms”, DWS). We focus on these latter two groups where there is disagreement between diagnosis and self-report symptoms. We do not report in detail on the three “agreement" categories in this paper as the paper is concerned with gaps between distress self-reported by respondents and access to professional support. Professional diagnosis in this instance is taken as a proxy for having accessed professional support (although we discuss later the extent to which this may or may not reflect access to appropriate support).

### Ethical approval

UKHLS has ethical approval from the University of Essex Ethics Committee. Additional ethical approval was not required for the secondary data analysis conducted in this study.

## Statistical analysis

Analysis was conducted using Stata v17 [28]. The most recent observation for each respondent from waves 6 to 10 were analysed cross-sectionally using multinomial regression. To reduce missing data, observations were pulled forward from earlier waves for diagnosis of clinical depression, religion, sexual orientation, and highest educational qualification achieved. Where the GHQ-12 score was missing in the most recent observation, the mean GHQ-12 score was calculated where two or more previous observations were available for the respondent (n = 345; 1%).

Two multinomial regression models were run using the mental health status variable as the outcome variable. The first model describes the risk of experiencing a mental health inequality for each protected characteristic (age, sex, marriage/civil partnership status, religion, ethnic group, sexual orientation and disability). The second model describes the risk of experiencing a mental health inequality for each protected characteristic when controlling for wider social and economic determinants of health (individual net income transformed using the inverse hyperbolic function, highest education qualification achieved, economic status and household size). To enable population level inference, the analyses were weighted using the cross-sectional weights provided by UKHLS. Relative Risk Ratios (RRR) and 95% confidence intervals (CI) were generated and reported for both models. In addition, predictive probabilities are presented in S2 and S3 Tables to aid interpretation.

## Results

### Sample characteristics

The sample characteristics are shown in Table 1 and are broadly similar to the UK population according to census data [29] although fewer people reporting their sexuality as LGB and slightly more people reporting a disability. A majority of respondents were women (55.7%), married or in civil partnership (53.4%), not religious (47.4%), heterosexual (96.3%), of White ethic background (79.6%) and without a disability (63.9). Most respondents were categorised as having no mental health need (77%—see S1 Table). Respondents’ GHQ-12 score used for analyses were reported in wave 10 (77.9%), wave 9 (10.1%), wave 8 (6.0%), and wave 7 (4.9%).

**Table 1:** Sociodemographic characteristics by sample, and mental health status category.

	Total n = 34,847 Freq (%)	UD n = 5,570 Freq (%)	DWS n = 448 Freq (%)	Agreement^Ϯ^ n = 28,829 Freq (%)
**Age Group**				
16–24	3,851 (11.1)	784 (14.1)	40 (8.9)	3,027 (10.5)
25–34	4,272 (12.3)	786 (14.1)	53 (11.8)	3,433 (11.9)
35–44	5,521 (15.8)	969 (17.4)	69 (15.4)	4,483 (15.6)
45–54	6,485 (18.6)	1,067 (19.2)	88 (19.6)	5,330 (18.5)
55–64	5,864 (16.8)	908 (16.3)	99 (22.1)	4,857 (16.8)
65–74	5,102 (14.6)	544 (9.8)	73 (16.3)	4,485 (15.6)
75+	3,752 (10.8)	512 (9.2)	26 (5.8)	3,214 (11.1)
**Sex**				
Men	15,434 (44.3)	2,063 (37)	172 (38.4)	13,199 (45.8)
Women	19,413 (55.7)	3,507 (63)	276 (61.6)	15,630 (54.2)
**Marital status**				
Married/civil p’ship	18,611 (53.4)	2,506 (45.0)	231 (51.6)	15,874 (55.1)
Unmarried/not in civil p’ship	16,236 (46.6)	3,064 (55.0)	217 (48.4)	12,995 (44.9)
**Religion**				
Not religious	16,501 (47.4)	2,790 (50.1)	248 (55.4)	13,463 (46.7)
Dominant religion	14,845 (42.6)	2,141 (38.4)	184 (41.7)	12,520 (43.4)
Minority religion	3,501 (10.0)	639 (11.5)	16 (3.6)	2,846 (9.9)
**Ethnicity**				
White British	27,740 (79.6)	4,280 (76.8)	411 (91.7)	23,049 (80.0)
Minority ethnic group	7,107 (20.4)	1,290 (23.2)	37 (8.3)	5,780 (20.0)
**Sexual orientation**				
Heterosexual	33,557 (96.3)	5,277 (94.7)	427 (95.3)	27,853 (96.6)
Lesbian, Gay, Bisexual	1,290 (3.7)	293 (5.26)	21 (4.7)	976 (3.4)
**Disability**				
No disability	22,256 (63.9)	2,852 (51.2)	190 (42.4)	19,214 (66.6)
Has disability	12,591 (36.1)	2,718 (48.8)	258 (57.6)	9,615 (33.4)
**Economic status**				
Employed	19,681 (56.5)	2,893 (52.0)	251 (56.0)	16,537 (57.4)
Unemployed	1,261 (3.6)	361 (6.5)	18 (4.0)	882 (3.1)
Retired	9,173 (26.3)	1,124 (20.2)	118 (26.3)	7,931 (27.5)
Other	4,713 (13.5)	1,191 (21.4)	61 (13.6)	3,461 (12.0)
**Highest education achieved**				
Higher education	15,269 (50.8)	2,613 (54.4)	181 (46.1)	12,475 (50.1)
Further education/professional	3,686 (12.3)	566 (11.8)	61 (15.5)	3,059 (12.30
High school	6,133 (20.4)	921 (19.2)	92 (23.4)	5,120 (20.6)
Other	4,993 (16.6)	707 (14.7)	59 (15.0)	4,227 (17.0)
	Total Mean (SD)	Undiagnosed distress Mean (SD)	DWS Mean (SD)	Agreement^Ϯ^
**Individual net income (£)**	1623.68 (1525.0)	1443.21 (1190.0)	1659.92 (1393.5)	1657.98 (1581.09)
**Mean household size**	2.85 (1.5)	2.92 (1.6)	2.51 (1.2)	2.85 (1.48)
**Mean GHQ-12 score**	1.82 (3.1)	7.22 (2.6)	0.62 (0.9)	0.79 (1.86)

Note: SD = standard deviation, GHQ-12 = 12-item General Health Questionnaire. UD = Undiagnosed distress, DWS = Diagnosis without self-report symptoms.

^Ϯ^Agreement refers to the combined three mental health status categories reflecting agreement between professional diagnosis and self-report symptoms.

### Are protected characteristics associated with undiagnosed distress?

The results of model 1 (Table 2) indicate decreased risk of experiencing UD for all age groups compared to those aged 16–24. A trend of decreasing risk was evident from age 35–44 until 75+ years of age, when risk slightly increased again. The RRR reduced from 0.97 (95% CI 0.84, 1.13) at age 35–44 years to 0.38 (95% CI 0.32, 0.44) at age 65–74 years, compared to those aged 16–24. The 75+ years age group had a risk of 0.44 (95% CI 0.37, 0.52). Risk ratios for ages 45–54, 55–64, 65–74 and 75+ were statistically significant. This also suggests those aged 16–24 years are at the greatest risk. Women (RRR = 1.49; 95% CI 1.38, 1.61), people who are unmarried or not in a civil partnership (RRR = 1.33; 95% CI 1.23, 1.44), LGB people (RRR = 1.42; 95% CI 1.19, 1.70) and people with disabilities (RRR = 2.76; 95% CI 2.55, 2.99) are all at significantly greater risk of experiencing UD, compared to the relevant reference group. Those affiliated with a dominant religion were subject to significantly decreased risk (RRR = 0.89; 95% CI 0.82, 0.97) while those affiliated with a minority religion had no increased or decreased risk (RRR = 1.00; 95% CI 0.85, 1.18), when compared to people with no religious affiliation. Predicted probabilities are provided in S2 Table.

**Table 2:** Relative Risk Ratios for protected characteristics and risk of undiagnosed distress.

	Model 1^a^ (n = 34,847)		Model 2^b^ (n = 30,067)	
Protected characteristics	RRR	95% CI	RRR	95% CI
**Age Group (Ref: 16–24)**				
25–34	0.90	0.77, 1.05	1.06	0.90, 1.26
35–44	0.97	0.84, 1.13	**1.22**	**1.03, 1.44**
45–54	**0.82**	**0.71, 0.94**	0.99	0.83, 1.16
55–64	**0.70**	**0.60, 0.81**	0.85	0.75, 1.03
65–74	**0.38**	**0.32, 0.44**	**0.52**	**0.41, 0.66**
75+	**0.44**	**0.37, 0.52**	**0.64**	**0.50, 0.83**
**Sex**				
Women	**1.49**	**1.38, 1.61**	**1.45**	**1.33, 1.57**
**Marital status (Ref: Married or civil partnership)**				
Unmarried/not in civil partnership	**1.33**	**1.23, 1.44**	**1.25**	**1.13, 1.37**
**Religion (Ref: not religious)**				
Dominant religion	**0.89**	**0.83, 0.97**	**0.90**	**0.83, 0.98**
Minority religion	1.00	0.85, 1.18	0.98	0.82, 1.16
**Ethnicity (Ref: White British)**				
Minority ethnic group	1.10	0.98, 1.24	1.05	0.93, 1.19
**Sexual orientation (Ref; Heterosexual)**				
Lesbian, Gay, Bisexual	**1.42**	**1.19, 1.70**	**1.41**	**1.16, 1.70**
**Disability (Ref: No disability)**				
Has disability	**2.76**	**2.55, 2.99**	**2.51**	**2.30, 2.73**
**Social and Economic Determinants**				
Individual net income	-	-	0.99	0.98, 1.02
**Economic Activity (Ref: Employed)**				
Unemployed	-	-	**2.35**	**1.91, 2.80**
Retired	-	-	0.98	0.83, 1.15
Other	-		**1.87**	**1.66, 2.12**
**Highest education achieved (Ref: Higher education)**				
Further education or professional training	-	-	0.89	0.78, 1.01
High school	-	-	**0.80**	**0.72, 0.89**
Other	-	-	**0.82**	**0.72, 0.93**
**Household size**	-	-	0.98	0.94, 1.02

Note: RRR = Relative Risk Ratio. CI = Confidence Interval.– = variable was not included in the model. Statistically significant results are highlighted in bold text. ^a^ unadjusted model. ^b^ model adjusted for individual net income, economic activity, educational attainment, and household size.

In model 2, which controlled for social and economic variables, unemployment and “other” economic status were associated with a significant increase in risk, compared to being employed. Having a high school education or “other” as the highest educational qualification achieved was associated with a significant decrease in risk, compared to those with higher education qualifications.

When controlling for social and economic variables, the relative risk for the 45–54 and 55–64 age groups became non-significant, while risk became significant in the 35–44 age group (RRR = 1.22; 95% CI 1.03, 1.44). This suggests risks in these age groups are impacted by education and economic status. The risk ratios were also attenuated slightly for the 65–74 age group (RRR = 0.52; 95% CI 0.41, 0.66) and the 75+ age group (RRR = 0.64; 95% CI 0.50, 0.83) compared to model 1, again suggesting that education and economic status impact on inequalities relating to age, although these impacts change over the life course. The magnitude of risk associated with being a woman (RRR = 1.45; 95% CI 1.33, 1.57), not being married or in a civil partnership (RRR = 1.25; 95% CI 1.13, 1.37), being LGB (RRR = 1.41; 95% CI 1.16, 1.70) and living with a disability (RRR = 2.51; 95% CI 2.30, 2.73), decreased and remained significant. These findings suggest the magnitude of risk is related to education and economic status, and that the strength of these relationships may vary by protected characteristic.

### Are protected characteristics associated with diagnosis without self-report symptoms

The mental health status category DWS referred to cases where there was no recent evidence of psychological distress (GHQ-12 caseness score of between 0–3 in all waves included) but the respondent has a history of mental health diagnosis suggesting the diagnosis was questionable or potentially unsupported. The 75+ age group had a significantly reduced risk of being in this category compared to the 16–24 age group (RRR = 0.32; 95% CI 0.17, 0.63). None of the other risk ratios associated with age were significant (Table 3).

**Table 3:** Relative Risk Ratios for protected characteristics and risk of diagnosis without self-report symptoms.

	Model 1^a^ (n = 34,847)		Model 2^b^ (n = 30,067)	
Protected characteristics	RRR	95% CI	RRR	95% CI
**Age Group (Ref: 16–24)**				
25–34	1.04	0.59, 1.82	1.08	0.59, 1.95
35–44	1.17	0.68, 1.99	1.32	0.75, 2.33
45–54	1.12	0.65, 1.91	1.05	0.60, 1.84
55–64	1.03	0.60, 1.77	0.94	0.55, 1.65
65–74	0.64	0.37, 1.10	0.56	0.26, 1.17
75+	**0.32**	**0.17, 0.63**	**0.34**	**0.15, 0.79**
**Sex**				
Women	**1.38**	**1.08, 1.76**	**1.44**	**1.11, 1.86**
**Marital status (Ref: Married or civil partnership)**				
Unmarried/not in civil partnership	1.13	0.88, 1.45	0.91	0.70, 1.20
**Religion (Ref: not religious)**				
Dominant religion	0.94	0.72, 1.21	0.96	0.73, 1.27
Minority religion	1.07	0.46, 2.50	1.10	0.45, 2.69
**Ethnicity (Ref: White British)**				
Minority ethnic group	**0.34**	**0.20, 0.61**	**0.36**	**0.20, 0.66**
**Sexual orientation (Ref; Heterosexual)**				
Lesbian, Gay, Bisexual	1.50	0.84, 2.69	1.53	0.86, 2.71
**Disability (Ref: No disability)**				
Has disability	**3.61**	**2.80, 4.66**	**3.48**	**2.64, 4.59**
**Social and Economic Determinants**				
Individual net income	-	-	0.99	0.91, 1.07
**Economic Activity (Ref: Employed)**				
Unemployed	-	-	1.70	0.86, 3.38
Retired	-	-	1.01	0.62, 1.66
Other	-	-	1.43	0.96, 2.15
**Highest education achieved (Ref: Higher education)**				
Further education or professional training	-	-	**1.53**	**1.07, 2.17**
High school	-	-	1.06	0.77, 1.45
Other	-	-	0.65	0.93, 1.30
**Household size**			**0.84**	**0.74, 0.95**

Note: RRR = Relative Risk Ratio. CI = Confidence Interval.– = variable was not included in the model. Statistically significant results are highlighted in bold text. ^a^ unadjusted model. ^b^ model adjusted for individual net income, economic activity, educational attainment, and household size.

Women had an increased risk of being in this category compared with men (RRR = 1.38; 95% CI 1.09, 1.76), as did people with a disability compared with people without a disability (RRR = 3.61; 95% CI 2.80, 4.66). People from a minority ethnic group had a third of the risk compared with White British people (RRR = 0.35; 95% CI 0.20, 0.61), indicating White British people are at greater risk of DWS.

In model 2, which controlled for social and economic variables, the only variable associated with significant increase to risk of having a questionable diagnosis was further/professional educational qualification as the highest educational attainment (RRR = 1.52; 95% CI 1.07, 2.17). Increased household size was associated with a reduced risk (RRR = 0.84; 95% CI 0.74, 0.95). When controlling for social and economic variables the risk of being in this category remained significant for the 75+ year age group with only a slight increase in magnitude (RRR = 0.34; 95% CI 0.15, 0.79). This suggests that for the oldest adults, education and household size have minimal impact on the already low risk of diagnosis without symptoms. As with UD, these findings suggest the association with social and economic variables and risk of DWS may vary over the life course. The level of risk was attenuated for people with a disability (RRR = 3.48; 95% CI 2.64, 4.59), which combined with the reduced risk associated with household size, suggests that living with others may reduce risk of having a questionable diagnosis for people with disability. The significant risk of DWS increased slightly for women (RRR = 1.44; 95% CI 1.11, 1.86) and people from a minority ethnic group (RRR = 0.36; 95% CI 0.20, 0.66), however, the latter still had reduced risk overall. Inequalities in these groups may be driven by level of educational attainment and household size. Predicted probabilities are provided in S3 Table.

## Discussion

The first aim of this study was to investigate whether protected characteristics are associated with having UD or having a DWS (a questionable or unsupported diagnosis). These two categories make up approximately 20% of the overall sample with about 80% of the overall sample showing agreement between their self-report symptoms and their diagnostic status (see Table 1). This indicates that approximately a fifth of the population may be either under- or over-treated in terms of mental distress. Focusing on these two groups of interest, the results revealed differences in the magnitude of risk for each of the protected characteristics investigated. In both mental health status categories, the largest discrepancy in risk was evident between people with or without a disability, and to a lesser extent between men and women with women and people with a disability being more ‘at risk’. LGB people were also more at risk than heterosexual people of having UD; while people from ethnic minority backgrounds had considerably reduced risk (meaning people from white backgrounds were more at risk). The age group with the largest risk of having UD was 16–24-year-olds while 35-44-year-olds were most at risk of having a questionable diagnosis (DWS).

Given that women and people with a disability appear to have higher risk for UD and DWS, it appears that the mental health needs of people in these groups may be poorly understood in a number of ways, for example their social difficulties may be more likely to be medicalised and their mental health difficulties may be more difficult to identify in health services. The considerably higher risk for people with a disability experiencing UD and having a questionable diagnosis (without self-report symptoms) fits with findings from studies that show that both society and mental health services are designed around patriarchal and ableist concepts of normality [30]. It is suggested that mental health services need to work to become much more attuned to the needs of people with disabilities and design services in partnership with people with disabilities. Furthermore, detailed analysis could help to identify particular types of disability at greatest disadvantage, for example people living with hearing and/or vision impairments. These improvements will be challenging given the lack of resource in NHS services [31]. Despite commitments from the UK Government to increase funding for mental health services [2] a disparity between mental health needs and policies and investment that support the mental health of people with protected characteristics is still evident.

UD as defined in this study implies that access has been inadequate in terms of “availability of services that are timely, appropriate, easy to get to and use, and sensitive to user choice and need” [3]. It could indicate that mental health support has not been sought or that support has been sought but the health professional did not identify the issues as a mental health condition. Undiagnosed need can be driven by factors such as lack of appropriate services (attuned to different needs of different groups); fear of stigma (particularly where a group already experiences discrimination based on one or more characteristic); fear that medication will be the only treatment offered combined with concerns about addictive nature of medication [32,33]; or pressure from self or others to follow cultural norms and roles (e.g. LGB people may not seek treatment because of heteronormative attitudes in healthcare).

The consequences of living with an untreated mental health condition can lead to long-term physical, social and occupational disability and premature mortality [34] and can mean that difficulties escalate in severity leading to greater likelihood of crisis admissions at a later point and/or criminalisation. Alternatively, undiagnosed need may indicate a preference or tendency to seek and receive support at times of stress or distress from within community or social networks rather than from health services. Given the latter possibility, we cannot assume UD is entirely unsupported but that mental health needs are not being identified and supported by health services. It is possible that community resources can and are meeting the needs of some people in distress. The finding that people belonging to religious or ethnic minority groups are not at increased risk of UD may point to this, but this would need further research.

DWS as defined in this analysis may suggest a tendency for health professionals to move quickly to mental health diagnosis rather than explore underlying issues in some groups, which could relate to social or economic stressors, loneliness or physical health problems. Social problems including loneliness are accountable for approximately 20% of GP appointments [35], and both LGB people and people with disability are more likely to experience loneliness and social isolation [36–38]. GPs report that they do not feel equipped to manage the impact of loneliness on patients’ mental health and may over-medicalise leading to increased stigma and difficulty talking about loneliness and social difficulties in appointments [39].

Study findings indicate the age group most at risk for DWS (with the risk increasing when taking social and economic factors into account) are 35-44-year-olds which could be accounted for by this age group having greater social stressors than other age groups, such as relatively lower income and high work pressure compared to older groups but with greater responsibilities than younger cohorts arising from responsibility for children and/or caring for ageing parents. These social and economic stressors could be mis-diagnosed as mental illness, which could be explored further through other forms of analyses. Further analyses could also explore whether temporal, reactive difficulties such as relationship breakdown or bereavement being mis-diagnosed may account for diagnoses without symptoms being more likely in this age group. In addition, the analyses revealed that the oldest age category had the lowest risk of having an unsupported diagnosis. This finding may be due to generational differences in attitudes towards mental health and seeking support. Research has shown older adults in the UK are less likely to disclose emotional difficulties and often regard adversity as just a normal aspect of life to be tolerated [40]; this could mean that older people are less likely to report social problems to their GP and therefore less likely to have social problems mis-diagnosed as mental illness.

The second aim of the study was to examine whether social and economic circumstances influence potential relationships between protected characteristics and risk of discrepancies between professional diagnosis and self-reported distress. Social and economic circumstances appear to have more influence on the risk UD than for DWS and that impact is most evident in terms of age such that relative risk ratios increased for all age groups (in reference to 16-24-year-olds). It is possible that employment and income for the over 25s influence inequalities in accessing mental health care because increasing focus on financial and family responsibilities may prevent over 25s accessing healthcare for their own needs.

When taking account of social and economic circumstances, the risk ratio for disability decreased both in the UD and the DWS category. This suggests that social and economic circumstances, particularly employment, may moderate the disadvantages faced by people with disabilities and that employment status may play a role in the lack of appropriate support for social and psychological needs of disabled people. This should be explored in more detail by analysing the relationship between employment status and mental health among people with disability. For instance, in the UK, there is a disability employment gap of 28.8% and data indicate a higher rate of redundancies for employees with disability compared with employees without disability during the coronavirus pandemic [41]. It is possible that employment policy requires more radical overhaul to address possible ongoing discrimination in employment practices and inadequate adjustments for people with disabilities. An evaluation of the Disability Confident Scheme conducted by the Department for Work and Pensions [42] indicated that less than half of employers signed up to the scheme had employed a person with a disability, long term health or mental health condition, since joining.

Household size was associated with reduced risk of DWS. This supports our earlier argument that social issues such as loneliness may be driving diagnosis without symptoms. Again, this proposition is also supported by existing evidence of increased risk of loneliness to people living with a disability and our finding that risk of DWS reduced for people with disability when controlling for household size. Further, in the current study sample, 24% of respondents living with a disability reported living alone, whilst only 12% of respondents without a disability reported living alone (results not shown).

### Clinical implications

This study highlights the need for mental health services to develop preventive services and interventions that consider the impact of protected characteristics and the developmental stages of the individuals at risk. The main findings show for example that late adolescence and young adulthood is a particularly vulnerable window in terms of mental health undiagnosed needs, perhaps due to the developmental tasks and social pressures experienced at this age and the lack of services attuned to these needs [43–45].

Similarly, LGB people report health professionals not understanding how their sexuality impacts on mental and physical health issues [9,10]. Mental health professionals and services need to develop awareness of the implications of protected characteristics on presentation of symptoms, diagnosis and treatment. In particular, people with disabilities are evidently at greatest disadvantage and in service planning and development it seems critical that they are involved and represented at all levels in order that their needs can be more fully understood and taken into account.

Approaches may include an overhaul of education and training for healthcare professionals emphasising psychosocial approaches; increasing social and community (non-medical) resources for mental health support co-designed with diverse communities especially those currently disadvantaged; stricter enforcement of minimum accessibility standards for healthcare providers (e.g. the UK’s Accessible Information Standard); and diversification of health and social care workforce (particularly those working with mental distress) to be representative of all groups including those at greater risk of health inequalities. Currently, the NHS focus on workforce diversification is on ethnic minorities [46], however, our research highlights that other groups also require better representation to ensure equal and adequate mental health care, in particular people with disabilities and LGB people.

This and similar research help to highlight systemic issues in the healthcare system that facilitate inequalities. However, focus on protected characteristics alone can obscure the role of socio-economic factors. Our analysis indicates that these may be a significant determinant, particularly employment status. Socio-economic status is not a protected characteristic, yet it has a major impact on mental health, intersects with and drives other disadvantages, and so should be given greater attention in healthcare policies seeking to address inequalities.

### Strengths and limitations

The strengths of the study include the use of the nationally representative dataset and large sample size. It is also the first study that the researchers are aware of that investigates mental health care inequalities in relation to UD and DWS, providing a new lens through which to consider inequalities. Weaknesses of the study include the use of the GHQ-12 to measure mental health which is not a diagnostic tool. Further, GHQ-12 scores may be affected by temporal issues and circumstances, which are not indicative of a chronic mental health disorder. Self-report mental health data is also subject to social desirability bias resulting in mental health conditions being under-reported. Participants also reported their health diagnoses, which has been criticised, for example events may be under reported if they have occurred over 6 months prior to questioning [47]. The question asking if participants previously had a diagnosis of any nervous, psychiatric or emotional problem occurred in Wave 10 (the last timepoint included). Therefore those diagnosed within 6 months prior to Wave 10 may have been more likely to report the diagnosis relative to those diagnosed prior to this. This would lead to inconsistencies such as a conservative estimate of diagnosis without symptoms. Alternatively, the diagnosis without symptoms category may be over-estimated given that it is possible some individuals could have received a correct diagnosis, entered treatment and recovered between two waves of data collection so that the symptoms were never picked up in UKHLS and the individuals never relapsed across the 5 years of data included. These limitations could potentially be addressed through access to administrative data as a gold standard measure of health service use. However this can also be problematic as clinical databases may be inaccurate or incomplete. In *Understanding Society*, linkage to routinely collected health service usage data is not fully available and rates of consent to data linkage are lower in those with poorer health and in minority ethnic groups [48] which would have served to bias our analyses.

Factors such as regional policies and identities may influence mental health inequalities and therefore magnitude and direction of risk may differ between regions of the UK. Pre-pandemic data was used to avoid the impact of the coronavirus pandemic on population level mental health confounding the findings. As such, the findings presented in this study may not accurately reflect the post-pandemic magnitude of risk of mental health inequalities for people with certain protected characteristics that are still being affected by societal changes, for example, people with sensory impairment and young adults affected by school closures [49] and further analyses will be needed to examine the longer term impacts of the pandemic. The categorisation of populations into binary variables may have masked nuances in the data, such as differences between minority ethnic groups or religious affiliation. As this study employed cross-sectional analysis, it did not look at changes in trends or within individuals over time.

## Conclusion

This is the first analysis of a representative UK sample to examine mental health inequality across protected characteristics, taking into account both UD as well as where diagnosis is made in the absence of self-report symptoms. Medicalisation of social problems may be one possible explanation for the latter. The findings indicate that in the UK adult general population, having a disability or being female are associated with increased risk of having both UD and DWS; being LGB is associated with increased risk of UD. Increasing age was associated with reduced risk of having UD and DWS. The most significant finding is that people with disabilities have considerably greater risk of both UD and having a questionable or unsupported diagnosis compared to people without a disability and the risk is much higher than for other protected characteristics. Other groups experiencing disadvantage to a lesser extent are young adults, women and LGB people. Further analysis would be required to understand which additional characteristics predict which people in these groups are more likely to experience UD or DWS, and where these risk factors intersect. Further analysis should investigate whether there is cumulative increased or decreased risk of experiencing a mental health inequality with additional protected characteristics and which groupings of characteristics experience the greatest risk. Longitudinal research could also be used to explore changes and trends within and across groups over time.

Previous studies of mental health in the UK suggest there may be regional differences in the populations at greatest risk of common mental disorders [1]. Whilst our national level analysis has highlighted the presence of mental health inequalities by protected characteristic, a regional version of this analysis would be beneficial to inform localised mental health care policies and practices. It is also critical to examine the role of socio-economic circumstances, employment in particular, to establish the way in which this may moderate risk over time, for example by analysing the impact of becoming unemployed or employed.

Additional research is essential to inform local and national policy development and healthcare strategy to address inequalities by improving access to mental health care both in health services and community settings.

## Supporting information

S1 Table Sociodemographic characteristics by mental health category. (DOCX)

S2 Table Predictive margins by mental health group (model 1). (DOCX)

S3 Table Predictive margins by mental health group (model 2). (DOCX)
