# Supplementary material for: Are protected characteristics associated with mental health care inequalities in the adult UK general population? a cross-sectional study
Source: PLoS One. 2024 Aug 6;19(8):e0308279. doi: 10.1371/journal.pone.0308279 (PMC11302902; doi:10.1371/journal.pone.0308279)
Supplement: S1 Table — (DOCX) [file pone.0308279.s001.docx]

Supplementary Table S1. Sociodemographic characteristics by mental health category

|  | No evidence of psychological distress  n (%) | Evidence of psychological distress  n (%) | No current evidence of psychological distress  n (%) |
| --- | --- | --- | --- |
| **No. in category** | 26,884 (77) | 1,156 | 789 |
| **Age Group** |  |  |  |
| 16-24 | 2,845 (10.6) | 119 (10.3) | 63 (8.0) |
| 25-34 | 3,134 (11.7) | 183 (15.8) | 116 (14.7) |
| 35-44 | 4,136 (15.4) | 203 (17.6) | 144 (18.3) |
| 45-54 | 4,911 (18.3) | 243 (21.0) | 176 (22.3) |
| 55-64 | 4,438 (16.5) | 242 (20.9) | 177 (22.4) |
| 65-74 | 4,290 (16.0) | 112 (10.5) | 83 (10.5) |
| 75+ | 3,130 (11.6) | 54 (3.8) | 30 (3.8) |
| **Sex** |  |  |  |
| Men | 12,628 (47.0) | 325 (28.1) | 246 (31.2) |
| Women | 14,256 (53.0) | 831 (71.9) | 543 (68.8) |
| **Marital status** |  |  |  |
| Married/civil p’ship | 15,078 (56.1) | 428 (37.0) | 368 (46.6) |
| Unmarried/not in civil p’ship | 11,806 (43.9) | 728 (63.0) | 421 (53.4) |
| **Religion** |  |  |  |
| Not religious | 12,389 (46.1) | 646 (55.9) | 428 (54.2) |
| Dominant religion | 11,776 (43.8) | 419 (36.2) | 325 (41.2) |
| Minority religion | 2,719 (10.1) | 91 (7.9) | 36 (4.6) |
| **Ethnicity** |  |  |  |
| White British | 21,370 (79.5) | 979 (84.7) | 700 (88.7) |
| Diverse ethnic background | 5,514 (20.5) | 177 (15.3) | 89 (11.3) |
| **Sexual orientation** |  |  |  |
| Heterosexual | 26,076 (97.0) | 1,049 (90.7) | 728 (92.3) |
| Lesbian, Gay, Bisexual | 808 (3.0) | 107 (9.3) | 61 (7.7) |
| **Disability** |  |  |  |
| No disability | 18,773 (69.8) | 191 (16.5) | 250 (31.7) |
| Has disability | 8,111 (30.2) | 965 (83.5) | 539 (68.3) |
| **Economic status** |  |  |  |
| Employed | 15,618 (58.1) | 462 (40.0) | 457 (57.9) |
| Unemployed | 739 (2.7) | 104 (9.0) | 39 (4.9) |
| Retired | 7,595 (28.3) | 192 (16.6) | 144 (18.3) |
| Other | 2,915 (10.8) | 397 (34.3) | 149 (18.9) |
| **Highest education achieved** | |  |  |
| Higher education | 11,745 (43.7) | 410 (35.5) | 320 (40.6) |
| Further education/professional | 2,802 (10.4) | 161 (13.9) | 96 (12.2) |
| High school | 4,716 (17.5) | 20 (19.5) | 154 (19.5) |
| Other | 3,917 (14.6) | 189 (15.3) | 121 (15.3) |
|  | **Mean (SD)** | **Mean (SD)** | **Mean (SD)** |
| **Individual net income (£)** | 1675 (1609) | 1328 (1071) | 1541 (1100) |
| **Mean household size*** | 2.86 (1.49) | 2.63 (1.45) | 2.58 (1.34) |
| **Mean GHQ-12 score*** | 9.10 (2.81) | 22.85 (5.77) | 10.88 (3.06) |

Note: SD = standard deviation, GHQ-12 = 12-item General Health Questionnaire.
