# Supplementary material for: Are protected characteristics associated with mental health care inequalities in the adult UK general population? a cross-sectional study
Source: PLoS One. 2024 Aug 6;19(8):e0308279. doi: 10.1371/journal.pone.0308279 (PMC11302902; doi:10.1371/journal.pone.0308279)
Supplement: S2 Table — (DOCX) [file pone.0308279.s002.docx]

Supplementary Table S2: Predictive margins by mental health group (model 1)

|  | Evidence of psychological distress  Mean probability (SE) | Undiagnosed distress  Mean probability (SE) | Diagnosis without self-report symptoms  Mean probability (SE) | No evidence of psychological distress  Mean probability (SE) | No current evidence of psychological distress  Mean probability (SE) |
| --- | --- | --- | --- | --- | --- |
| **Age Group** |  |  |  |  |  |
| 16-24 | 0.023  (0.003) | 0.206  (0.01) | 0.013  (0.003) | 0.735  (0.01) | 0.022  (0.004) |
| 25-34 | 0.029  (0.003) | 0.186  (0.01) | 0.014  (0.003) | 0.738  (0.01) | 0.033  (0.004) |
| 35-44 | 0.027  (0.003) | 0.199  (0.001) | 0.015  (0.002) | 0.730  (0.009) | 0.029  (0.003) |
| 45-54 | 0.022  (0.002) | 0.174  (0.006) | 0.015  (0.002) | 0.761  (0.007) | 0.026  (0.002) |
| 55-64 | 0.019  (0.002) | 0.155  (0.006) | 0.014  (0.002) | 0.790  (0.007) | 0.022  (0.002) |
| 65-74 | 0.009  (0.001) | 0.093  (0.005) | 0.010  (0.001) | 0.879  (0.005) | 0.010  (0.001) |
| 75+ | 0.004  (0.001) | 0.108  (0.005) | 0.005  (0.001) | 0.880  (0.006) | 0.004  (0.001) |
| **Sex** |  |  |  |  |  |
| Men | 0.012  (0.001) | 0.133  (0.004) | 0.011  (0.001) | 0.831  (0.004) | 0.014  (0.001) |
| Women | 0.023  (0.002) | 0.181  (0.004) | 0.013  (0.001) | 0.761  (0.004) | 0.022  (0.001) |
| **Marital status** |  |  |  |  |  |
| Married/civil p’ship | 0.012  (0.001) | 0.140  (0.003) | 0.012  (0.001) | 0.820  (0.004) | 0.016  (0.001) |
| Unmarried/not in civil p’ship | 0.022  (0.002) | 0.175  (0.004) | 0.012  (0.001) | 0.771  (0.004) | 0.020  (.0002) |
| **Religion** |  |  |  |  |  |
| Not religious | 0.017  (0.001) | 0.163  (0.004) | 0.012  (0.001) | 0.790  (0.004) | 0.017  (0.001) |
| Dominant religion | 0.015  (0.001) | 0.148  (0.004) | 0.012  (0.001) | 0.805  (0.004) | 0.020  (0.002) |
| Minority religion | 0.026  (0.005) | 0.163  (0.011) | 0.013  (0.005) | 0.787  (0.013) | 0.012  (0.003) |
| **Ethnicity** |  |  |  |  |  |
| White British | 0.017  (0.001) | 0.155  (0.003) | 0.015  (0.001) | 0.795  (0.003) | 0.019  (0.001) |
| Diverse ethnic background | 0.012  (0.002) | 0.172  (0.008) | 0.005  (0.001) | 0.800  (0.008) | 0.012  (0.002) |
| **Sexual orientation** |  |  |  |  |  |
| Heterosexual | 0.016  (0.001) | 0.155  (0.003) | 0.012  (0.001) | 0.800  (0.003) | 0.017  (0.001) |
| Lesbian, Gay, Bisexual | 0.032  (0.004) | 0.197  (0.014) | 0.016  (0.005) | 0.715  (0.016) | 0.040  (0.006) |
| **Disability** |  |  |  |  |  |
| No disability | 0.006  (0.001) | 0.115  (0.003) | 0.008  (0.001) | 0.862  (0.003) | 0.010  (0.001) |
| Has disability | 0.076  (0.003) | 0.231  (0.005) | 0.021  (0.002) | 0.627  (0.006) | 0.045  (0.003) |
