# Supplementary material for: Are protected characteristics associated with mental health care inequalities in the adult UK general population? a cross-sectional study
Source: PLoS One. 2024 Aug 6;19(8):e0308279. doi: 10.1371/journal.pone.0308279 (PMC11302902; doi:10.1371/journal.pone.0308279)
Supplement: S3 Table — (DOCX) [file pone.0308279.s003.docx]

Supplementary Table S3: Predictive margins by mental health group (model 2)

|  | Evidence of psychological distress Mean Probability (SE) | Undiagnosed distress  Mean probability (SE) | Diagnosis without self-report symptoms  Mean probability (SE)) | No evidence of psychological distress  Mean probability (SE | No current evidence of psychological distress  Mean probability (SE) |
| --- | --- | --- | --- | --- | --- |
| **Age Group** |  |  |  |  |  |
| 16-24 | 0.019  (0.003) | 0.170  (0.010) | 0.013  (0.003) | 0.771  (0.012) | 0.027  (0.005) |
| 25-34 | 0.027  (0.003) | 0.176  (0.010) | 0.014  (0.003) | 0.748  (0.011) | 0.036  (0.005) |
| 35-44 | 0.025  (0.003) | 0.196  (0.009) | 0.017  (0.003) | 0.730  (0.010) | 0.036  (0.005) |
| 45-54 | 0.021  (0.002) | 0.168  (0.007) | 0.014  (0.002) | 0.770  (0.008) | 0.027  (0.003) |
| 55-64 | 0.015  (0.002) | 0.151  (0.007) | 0.013  (0.002) | 0.801  (0.007) | 0.020  (0.002) |
| 65-74 | 0.010  (0.002) | 0.101  (0.007) | 0.008  (0.002) | 0.873  (0.008) | 0.008  (0.002) |
| 75+ | 0.004  (0.001) | 0.123  (0.010) | 0.005  (0.002) | 0.865  (0.010) | 0.003  (0.001) |
| **Sex** |  |  |  |  |  |
| Men | 0.011  (0.001) | 0.134  (0.004) | 0.010  (0.001) | 0.830  (0.004) | 0.015  (0.001) |
| Women | 0.022  (0.002) | 0.178  (0.004) | 0.014  (0.001) | 0.764  (0.004) | 0.023  (0.002) |
| **Marital status** |  |  |  |  |  |
| Married/civil p’ship | 0.013  (0.001) | 0.142  (0.004) | 0.013  (0.001) | 0.814  (0.004) | 0.018  (0.002) |
| Unmarried/not in civil p’ship | 0.019  (0.002) | 0.170  (0.004) | 0.011  (0.001) | 0.781  (0.005) | 0.019  (0.002) |
| **Religion** |  |  |  |  |  |
| Not religious | 0.016  (0.001) | 0.162  (0.004) | 0.012  (0.001) | 0.793  (0.004) | 0.017  (0.001) |
| Dominant religion | 0.014  (0.001) | 0.148  (0.004) | 0.012  (0.001) | 0.805  (0.005) | 0.022  (0.002) |
| Minority religion | 0.025  (0.005) | 0.158  (0.011) | 0.013  (0.006) | 0.791  (0.013) | 0.014  (0.004) |
| **Ethnicity** |  |  |  |  |  |
| White British | 0.017  (0.001) | 0.154  (0.003) | 0.014  (0.001) | 0.796  (0.003) | 0.020  (0.001) |
| Diverse ethnic background | 0.011  (0.002) | 0.164  (0.008) | 0.005  (0.001) | 0.808  (0.009) | 0.012  (0.002) |
| **Sexual orientation** |  |  |  |  |  |
| Heterosexual | 0.015  (0.001) | 0.154  (0.003) | 0.012  (0.001) | 0.801  (0.003) | 0.018  (0.001) |
| Lesbian, Gay, Bisexual | 0.033  (0.005) | 0.914  (0.014) | 0.017  (0.005) | 0.717  (0.017) | 0.040  (0.007) |
| **Disability** |  |  |  |  |  |
| No disability | 0.006  (0.001) | 0.119  (0.003) | 0.008  (0.001) | 0.857  (0.003) | 0.010  (0.001) |
| Has disability | 0.069  (0.004) | 0.223  (0.005) | 0.021  (0.002) | 0.641  (0.006) | 0.046  (0.003 |
| **Economic status** |  |  |  |  |  |
| Employed | 0.013  (0.001) | 0.142  (0.004) | 0.011  (0.001) | 0.815  (0.009) | 0.017  (0.002) |
| Unemployed | 0.049  (0.007) | 0.264  (0.019) | 0.015  (0.005) | 0.648  (0.022) | 0.024  (0.006) |
| Retired | 0.015  (0.002) | 0.139  (0.008) | 0.012  (0.002) | 0.815  (0.009) | 0.020  (0.003) |
| Other | 0.033  (0.003) | 0.229  (0.010) | 0.014  (0.003) | 0.704  (0.011) | 0.021  (0.003) |
| **Highest education achieved** |  |  |  |  |  |
| Higher education | 0.014  (0.001) | 0.170  (0.004) | 0.011  (0.001) | 0.789  (0.004) | 0.016  (0.001) |
| Further education/professional | 0.023  (0.003) | 0.150  (0.008) | 0.017  (0.003) | 0.787  (0.009) | 0.022  (0.003) |
| High school | 0.019  (0.002) | 0.139  (0.006) | 0.012  (0.008) | 0.810  (0.006) | 0.021  (0.002) |
| Other | 0.016  (0.002) | 0.142  (0.007) | 0.011  (0.002) | 0.811  (0.008) | 0.021  (0.003) |
| **Income (at median value £1302)** | 0.037  (0.001) | 0.162  (0.003) | 0.014  (0.001) | 0.762  (0.003) | 0.026  (0.001) |
